# From mechanical force to cellular response: the role of autophagy in orthodontic tooth movement

**DOI:** 10.3389/fphys.2026.1783943

**Published:** 2026-03-25

**Authors:** Yuan Dong, Yangyu Liu, Zhiqiang Hu, Chongxiao Qu

**Affiliations:** 1 Department of Pathology, Shanxi Provincial People’s Hospital, Shanxi Medical University, Taiyuan, Shanxi, China; 2 Department of Orthodontic, Hospital of Stomatology, Jilin University, Changchun, Jilin, China

**Keywords:** autophagy, orthodontic tooth movement, orthodontic-induced inflammatory root resorption, osteocytes, periodontal ligament cells

## Abstract

Orthodontic tooth movement (OTM) is a process responding to orthodontic forces, which converts mechanical stresses into biochemical events to reconstruct the periodontal ligament and alveolar bone. As a lysosome-dependent catabolic process induced by various cellular stress conditions, autophagy maintains the homeostasis of cells, tissues, and organs and is widely involved in different physiologic or pathologic processes in the tooth. Recently, accumulating studies have investigated the role of autophagy in OTM. In this review, we focus on the autophagy induced by orthodontic forces, discuss the multifaceted effects of autophagy in bone remodeling, inflammation, and complications in OTM, which may reveal the potential clinical implications of autophagy in OTM and provide potential therapeutic targets for future orthodontic clinical treatment.

## 1 Introduction

The demand for orthodontic treatment is rising as the general health and expectations of the population continue to improve. The objective of orthodontic treatment is to move teeth that have been misaligned to an appropriate position through the remodeling of the periodontium stimulated by orthodontic forces (Li et al., 2021b). Orthodontic tooth movement (OTM) is a fundamental requirement during any orthodontic treatment, and is enabled through a mediated cell/tissue mechanism performed by applying a force or a pair of forces on the dental elements (Cultrera et al., 2022). However, the mechanisms of OTM are still not completely understood. Exploring the underlying mechanisms of OTM is of utmost significance to shorten the duration of orthodontic treatment and reduce possible side effects.

Autophagy is a highly conserved, lysosome-dependent metabolic process by which cells degrade long-lived and macromolecular proteins as well as damaged organelles to tolerate starvation, clear incorrectly folded proteins, and repair impaired aging organelles (Raee et al., 2023; Wang et al., 2024). Autophagy is therefore crucial in sustaining the homeostasis of cells, tissues, and organs (Levine and Kroemer, 2019). Dysregulated autophagic activity is linked to various human illnesses such as cancers and neurodegenerative disorders (Yang and Klionsky, 2020; Klionsky et al., 2021). The complicated implications of autophagy have been investigated intensively in human oral diseases such as periodontal disease, periapical lesions, oral candidiasis, and oral cancer (Tan et al., 2017; Jiang et al., 2020). Recently, increasing studies have investigated the functions of autophagy in OTM. Using the mouse/rat OTM model *in vivo* and cells subjected to orthodontic forces *in vitro*, researchers have pointed out the existence and significance of autophagy during OTM (Zhang et al., 2018; Chen et al., 2019; Li et al., 2020; Xu et al., 2021; Zheng et al., 2022; Gao et al., 2023; Shao et al., 2024). In this review, we focus on the autophagy induced by orthodontic forces and discuss the potential mechanisms. Then we analyze the functions of autophagy in bone formation and resorption, aseptic inflammation, and the complication of OTM, thereby revealing the potential clinical implications of autophagy in OTM.

## 2 Mechanobiology of OTM

### 2.1 Hypothetical theories for mechanisms of OTM

**Figure 1:**
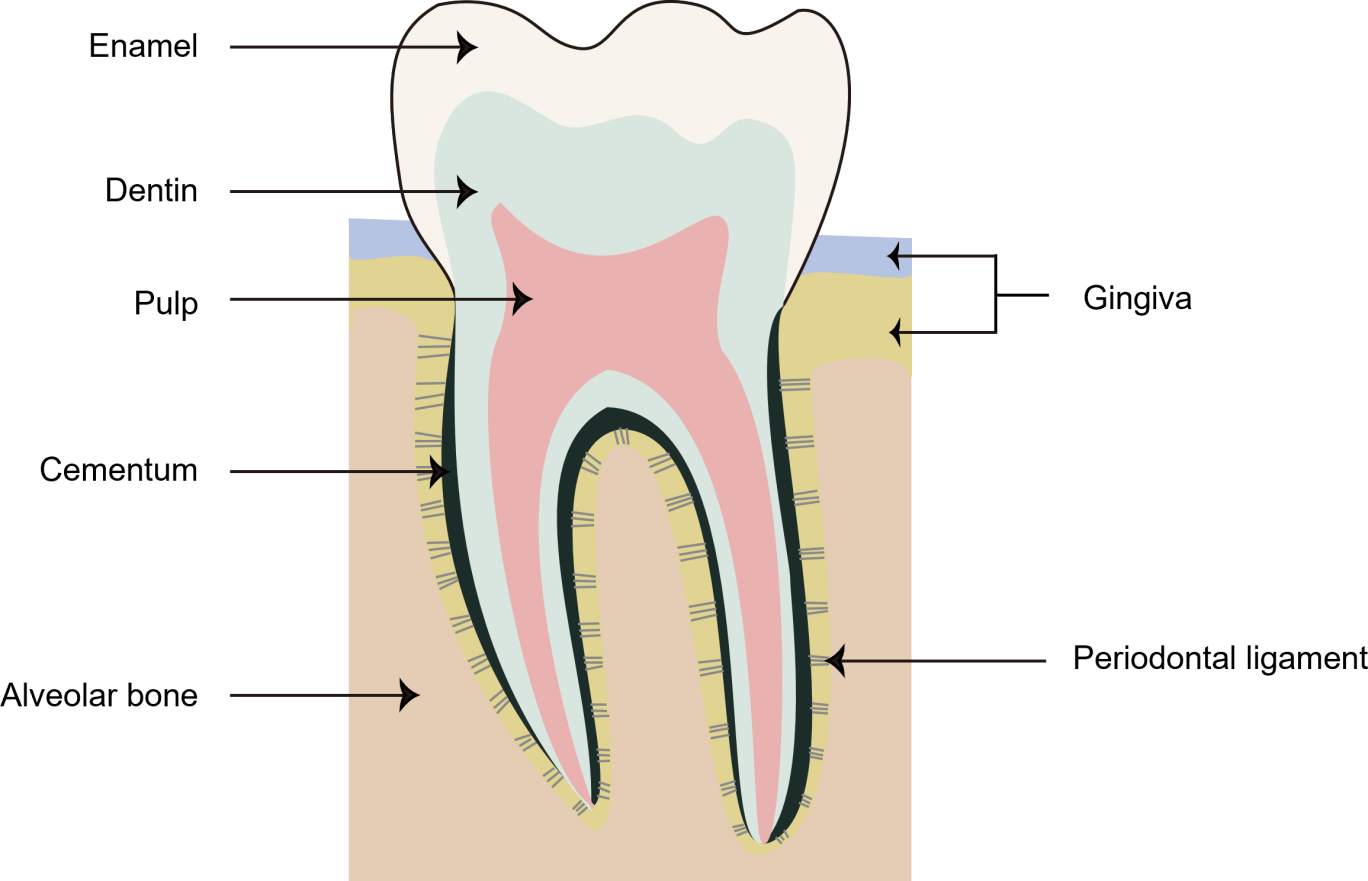
The structure of periodontium. The gingiva is the keratinized oral mucosa that attaches to the alveolar bone and creates a physical barrier to pathogens. PDL is a soft tissue between the cementum and the alveolar bone, providing support to the periodontium. The cementum is the outermost layer of hard tissue covering the dentin within the root portion of the teeth. The alveolar bone is a mineralized connective tissue that can hold the roots of teeth, absorbing and distributing occlusal forces generated by oral functions.

The periodontium is the tissue that supports and invests the teeth, comprising the gingiva, the periodontal ligament (PDL), the cementum, and the alveolar bone. These structures are categorized into the soft tissues (gingiva and PDL) and the hard tissues (cementum and alveolar bone) (**Figure 1**) (Nanci and Bosshardt, 2000). When appropriate orthodontic forces are applied, changes in the mechanical loading of biological systems result in strain, subsequently leading to cellular responses to adapt the system to the changed conditions (Henneman et al., 2008). The PDL and the alveolar bone undergo a series of reactions to change mechanical forces into molecular events during OTM (Masella and Meister, 2006).

The classic pressure-tension theory proposes that the application of orthodontic forces to the tooth site can generate the compression side and the tension side in the periodontium, and OTM is an outcome of bone resorption in the compression region and bone formation in the tension region (Ling et al., 2021; Yan et al., 2021).

Compared with the classical theory, the fluid flow hypothesis not only simply describes the bone remodeling during OTM. It focuses on how osteocytes and fibroblasts convert mechanical stress into biochemical events through complex molecular signaling networks. This hypothesis suggests that the process of the transduction from mechanical loadings to biological signals can be divided into four steps: (1) matrix strain and fluid flow, (2) cell strain, (3) cell activation and differentiation, and (4) tissue remodeling (Henneman et al., 2008; Li et al., 2021b).

#### 2.1.1 Matrix strain and fluid flow

Orthodontic forces cause the compression on the compression side and stretching on the tension side of PDL fibers. Both strains result in matrix deformation and fluid flow alteration (Li et al., 2021b). Fibroblasts in PDL and osteocytes in bone are mechanosensitive cells that sense strain and transduce mechanical stimuli into biochemical signals (Li et al., 2018). The porosity of the PDL allows the free fluid phase to redistribute within the periodontal space, which causes compression or stretching of collagen fibers surrounding cells in PDL, induces configurational changes of the Extracellular Matrix (ECM) proteins, and finally results in cell strains in PDL cells (PDLCs) (Li et al., 2021b; Maltha and Kuijpers-Jagtman, 2023). Furthermore, the external forces also result in a strain in the alveolar bone through the effect of fluid flow on osteocytes. Fluid flow applies shear stress to the bone ECM and cell membrane, thereby leading to cell strains in osteocytes (Henneman et al., 2008; Li et al., 2018).

#### 2.1.2 Cell strain

The transduction of strain from the ECM to PDL fibroblasts (PDLFs) is mediated by focal adhesion complexes (FACs). Through the cytoskeleton, FACs transmit mechanical stimuli from the ECM to the nucleus, where transcription factors activate gene expression (Maltha and Kuijpers-Jagtman, 2023). Other mechanosensitive ion channels, such as PIEZO1, also possess critical roles in mediating mechanotransduction (Schröder et al., 2023). The mechanism by which osteocytes sense, transfer, and respond to mechanical strain remains unclear. It is believed that osteocyte processes, integrins, and ion channels are involved in mechanotransduction (Murshid, 2017). Integrins, the main component of FACs, allow for the transduction of force from the ECM to the cell. The deformation of integrins by the mechanical stimuli may cause the transduction of mechanical signals that lead to changes in gene expression (Murshid, 2017; Geoghegan et al., 2019).

#### 2.1.3 Cell activation and differentiation

PDL consists of functionally differentiated cells like fibroblasts, cementoblasts, osteoblasts, osteoclasts, as well as undifferentiated mesenchymal stem cells, PDL stem cells (PDLSCs) (He et al., 2023). Activated by orthodontic forces, PDLCs regulate osteoclasts and osteoblasts by expressing some regulators such as Wnts, GDF15, asporin, and periostin (Ueda et al., 2016; Xu et al., 2017; Symmank et al., 2019; Ei Hsu Hlaing et al., 2020).Wnt pathways upregulate multiple osteogenic genes to promote differentiation (Ei Hsu Hlaing et al., 2020). GDF15 belongs to the transforming growth factor (TGF)-β/bone morphogenetic protein (BMP) superfamily, which integrates mechanobiological signals critical for skeletal development and bone homeostasis (Symmank et al., 2019). ASPN inhibits bone formation by directly binding to BMP-2 and suppressing TGF-β/Smad pathway (Ueda et al., 2016). Periostin is a multifunctional extracellular matrix protein, and may be associated with TGF-β, type I collagen and α-SMA expression (Xu et al., 2017).

PDLSCs have been shown to share characteristics with mesenchymal stem cells (MSCs). Both tension and compression can regulate the osteogenic differentiation of PDLSCs. Various mechanosensors and pathways, such as MAPK signaling, TGF-β/Smad, and Wnt/β-catenin are involved in these events (Huang et al., 2018). The receptor activator of nuclear factor-kappa B ligand (RANKL) is essential for osteoclastogenesis, and osteocytes are regarded as the critical source of RANKL in alveolar bone remodeling during OTM (Shoji-Matsunaga et al., 2017). There is a communication network mediated partly by sclerostin and Wnt proteins between PDLCs and osteocytes, synergistically regulating the differentiation of osteoclasts and osteoblasts (Odagaki et al., 2018; Ei Hsu Hlaing et al., 2020). In summary, complex regulatory networks are formed partially by PDLCs, osteocytes, osteoclasts, and osteoblasts to induce PDL and bone remodeling during OTM.

#### 2.1.4 Tissue remodeling

Under physiological conditions, the synthesis and degradation of the periodontal structures are at a low level to maintain tissue homeostasis. This balance is disturbed following the application of external forces, and increased remodeling of both PDL and alveolar bone results in tooth movement (Henneman et al., 2008). On the compression side, PDL is degraded to create space for tooth movement while new PDL tissue is simultaneously formed to maintain the attachment. On the tension side, PDL remodeling occurs after the fibers are stretched. New PDL matrixes containing type I collagen fibers are formed to maintain the PDL width and the attachment of the alveolar bone to the tooth (Li et al., 2021b). Besides, the coordination of osteoclasts responsible for bone resorption and osteoblasts for bone deposition provides the physiological basis of bone remodeling (Gong et al., 2023).

### 2.2 Inflammation in OTM

Based on the hypothesis, orthodontic loading alters fluid flow and develops regional hypoxia, which induce an aseptic inflammatory response devoid of bacteria. The aseptic inflammatory response caused by orthodontic forces promotes PDL and bone remodeling, which is indispensable for tooth movement (Li et al., 2018).

PDLCs and osteocytes are the most important cells that can induce the aseptic inflammatory response, secrete inflammatory factors, and thus regulate osteogenesis and osteoclastogenesis (Li et al., 2021b). Some viewpoints suggest that immune cells may play auxiliary roles in inflammation triggers (Li et al., 2021b). On the other hand, some studies reveal that activated immune cells play a pivotal role in OTM (Gao et al., 2022). For instance, macrophages can be activated into M1 or M2 polarization based on signals from the microenvironment (Fan et al., 2024). M1 macrophages mediate the inflammatory process, while M2 macrophages are responsible for the resolution of inflammation, wound healing, and tissue repair (Koudstaal et al., 2024). The accumulation of M1 macrophages after applying mechanical forces promotes OTM by tumor necrosis factor (TNF)-α (He et al., 2015b). M2-type macrophages do not significantly increase until a later stage of OTM and are important for the cessation of bone resorption and initiation of tissue repair (Wang et al., 2018). Besides, M1-type macrophages mediate inflammation by producing TNF-α, interleukins (IL)-1, and IL-6 (He et al., 2015b). These factors can stimulate osteoclast differentiation by inducing the release of RANKL and MCS-F (Tani-Ishii et al., 1999; Zhang et al., 2001; Liu et al., 2005).

Although inflammation is necessary for OTM and may be involved in accelerating OTM, unregulated or excessive inflammation is problematic and leads to tooth destruction (Li et al., 2018; Yamaguchi and Fukasawa, 2021). Elucidating the molecular events and investigating the regulatory factors of inflammation during OTM may provide a useful way to effectively regulate inflammation in clinical orthodontic treatment.

## 3 Molecular machinery of autophagy

Central to autophagy are a suite of autophagy-related genes (ATGs) and their encoded proteins (Amirian et al., 2025). The autophagy process is typically induced by various conditions of stress. Induction of autophagy results in the recruitment of ATGs to a specific subcellular location called the phagophore assembly site (PAS) and the nucleation of an isolation membrane that forms a cup-shaped structure called the phagophore (Dikic and Elazar, 2018). The Unc-51-like kinase (ULK) complex is the central regulator in the initiation of autophagy (Yamamoto et al., 2023). Then, the Class III PI3K complex (consisting of VPS34, Beclin 1, ATG14, and VPS15) is recruited by the ULK complex and generates PI3P, a crucial membrane signal for recruiting other ATGs, to promote phagophore nucleation (Amirian et al., 2025).

In phagophore expansion, two conjugation systems involving ubiquitin-like (UBL) proteins are prominently implicated: the ATG12–ATG5-ATG16L1 complex, and the ATG8/LC3 system (Parzych and Klionsky, 2014; Jacquet et al., 2021). Catalyzed by ATG7 and ATG3, LC3 is conjugated to membrane-associated phosphatidylethanolamine (PE) and then, anchored to the phagophore membrane by the ATG12-ATG5-ATG16L1 complex (Li et al., 2020). Thereby, LC3 is converted from a freely diffuse form (LC3-I) into a membrane-anchored, lipidated form (LC3-II), a widely used marker for autophagosome (Fujita et al., 2008; Dikic and Elazar, 2018). Cargo recognition proteins like p62 (SQSTM1) act as selective autophagy receptors, linking ubiquitinated cargo to LC3 on the autophagosome membrane for targeted degradation (Amirian et al., 2025). The expanding phagophore eventually mature and close to form a completed autophagosome. The double-membrane autophagosomes fuse with lysosome to form autolysosomes, transferring the sequestered material to the lysosomal compartment and degrading the cargo (Lőrincz and Juhász, 2020).

## 4 Activation mechanism of autophagy in OTM

Autophagy is essential for cellular homeostasis and stress adaptation. Emerging studies have confirmed the role of autophagy in physiologic or pathologic processes in the tooth, including tooth development, dental pulp aging, pulpitis, and periodontal disease (Yang et al., 2021). In recent years, the activation of autophagy in OTM has gained increasing attention.

### 4.1 Mechanical forces induce autophagy

OTM depends on appropriate sustained mechanical forces that activate biological reactions within the periodontium. When mechanical forces are applied, cells in PDL and the alveolar bone accept external mechanical stimuli, which activate autophagy.

#### 4.1.1 PDL and PDLCs

On the compression and tension sides of the PDL in the rat OTM model, IHC staining observed that the expression of Beclin-1 and LC3B (a specific isoform of the LC3 protein family) gradually increases over time, reaching the highest mean values before decreasing progressively, while p62 expression follows a completely different trend. Due to the sustained mechanical forces, the autophagy level is greater than its baseline level for a longer time (Xu et al., 2020). In the mouse OTM model, it is also observed that orthodontic compressive force increases the expression of autophagy-related proteins in PDL tissue (Chen et al., 2019).

During orthodontic treatment, the microenvironment of PDLCs changes, and initiates multiple signal transduction (Adnan et al., 2018; Singh et al., 2018; Yan et al., 2018; Onizuka and Iwata, 2019). Continuous stress in rats induces PDL remodeling following OTM. IHC observed that Beclin-1, LC3-II, and p62 are mainly expressed in the cytoplasm of PDLCs. The expression levels of Beclin-1 and LC3-II are significantly increased after 1 hour of orthodontic forces, while the opposite expression of p62 is observed, which indicates that autophagy is rapidly initiated after OTM (Wang et al., 2021). Similarly, human PDLCs exhibit more expression of autophagy-related proteins and autophagosomes under compressive force (Chen et al., 2019).

PDLSCs respond to orthodontic forces, maintaining periodontal homeostasis as well as periodontal and osseous remodeling through self-renewal, multidirectional differentiation, and immune regulation (Masella and Meister, 2006; Huang et al., 2018). In human PDLSCs under orthodontic tension force loading, the expression of ATG proteins increases (Zheng et al., 2022).

Excessive orthodontic forces increase the rate of cell death of PDLFs, whereas minimal forces may be too small to initiate tooth movement, both of which lead to a decelerated orthodontic treatment (Marchesan et al., 2011). Memmert et al. analyzed the influence of force magnitude on autophagy regulation and subsequently on cell death in human PDLFs (Memmert et al., 2019). PDLFs are treated with static tensile strain of low (3%, STSL) and high magnitudes (20%, STSH) that are confirmed to be realistic compared to the strain subjected to the PDL in the course of OTM, by chewing or grinding (Natali et al., 2004). They found that STS has regulatory effects on mRNA expression of multiple autophagy-associated targets. The autophagic flux is induced by STSH, while STSL has no significant effect on autophagosome formation, and the low magnitude of tensile strain seems to have cell-protective properties (Memmert et al., 2019). In addition to the force magnitude, the type of force also exhibits different regulatory effects on autophagy *in vitro* experiments. The mRNA expression of p62 is upregulated in PDLCs loading Cyclic tensile strain of low magnitude (CTSL), STSL, or STSH. However, STSL does not change p62 protein levels (Memmert et al., 2020).

#### 4.1.2 Osteocytes

Osteocytes sense mechanical loading and transduce signaling molecules to stimulate osteoclastic bone resorption, or osteoblastic bone formation (Seddiqi et al., 2023). In the alveolar bone of the mouse OTM model, immunofluorescence (IF) staining revealed that the number of LC3B-positive osteocytes is increased by 3-fold on the compression side (Li et al., 2020), and by 4-fold on the tension side (Xu et al., 2021). *In vitro*, mechanical compressive force evidently increases the mRNA and protein expression of autophagy markers and the number of autophagosomes in MLO-Y4 osteocytes under mechanical compressive force or mechanical tension (Li et al., 2020; Xu et al., 2021). These data suggest that autophagy in osteocytes is activated on both the compression and the tension side during OTM. Similarly, increased autophagic flux is observed in MLO-Y4 cells after exposure to compressive cyclic force (CCF) and fluid shear stress (FSS) (Zhang et al., 2018; Gao et al., 2023).

### 4.2 Activation mechanism

**Figure 2:**
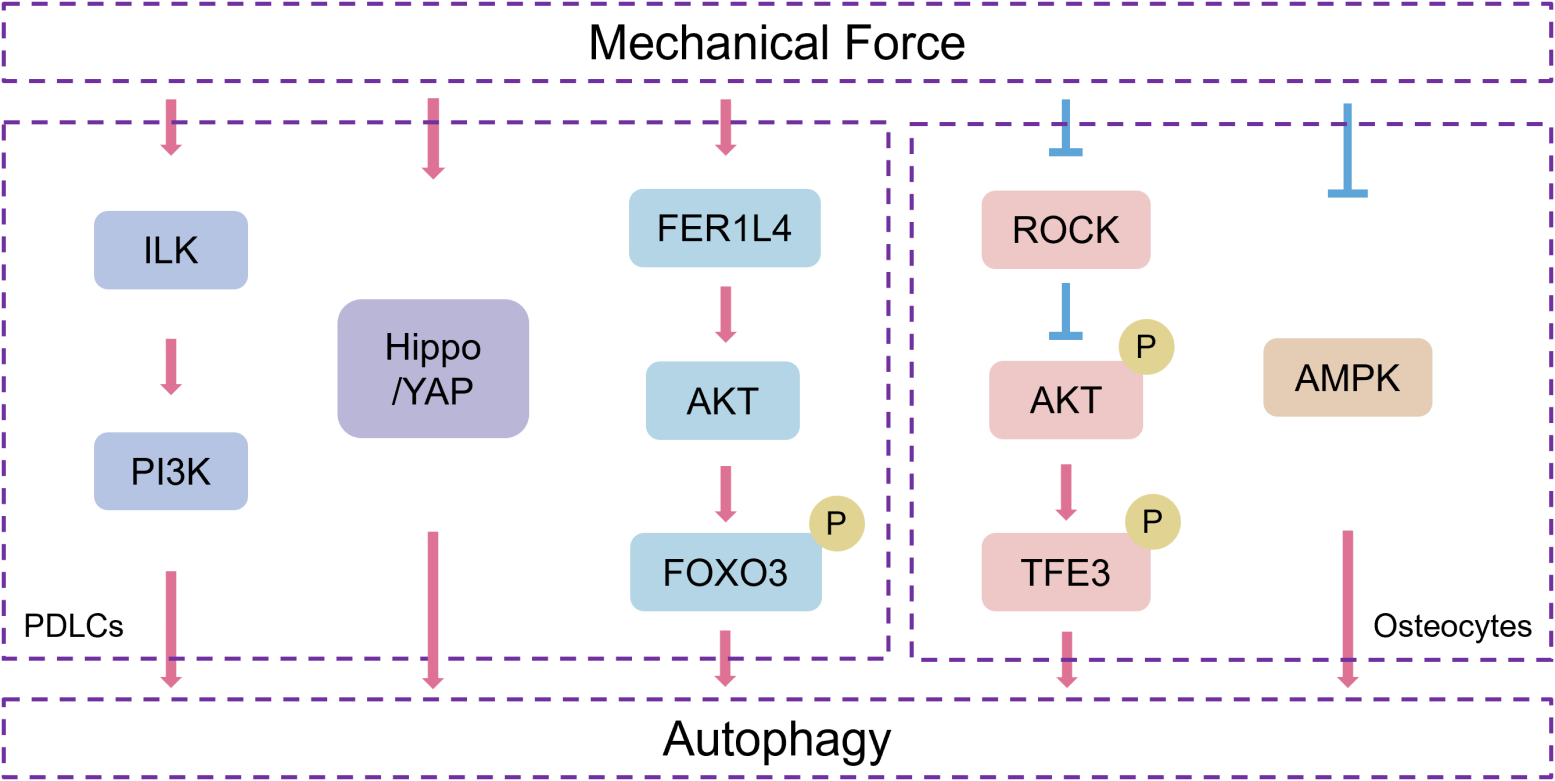
The related molecular signaling involved in mechanical force-induced autophagy in PDLCs and osteocytes during OTM.

Mechanical forces activate autophagy in cells in PDL and alveolar bone during OTM. However, the specific activation mechanism is still unclear. Several studies have preliminarily revealed that orthodontic forces may activate autophagy through related molecular signaling or by inflammatory factors (**Figure 2**).

#### 4.2.1 Molecular signaling

Static compressive stress (SCS) induces autophagy in human primary PDLCs, and ILK is involved in the process. After treatment with the specific inhibitor of PI3K, LY294002, the autophagy level and the expression of ILK of human PDLCs are no longer increased with stress loading, which suggests that SCS-induced autophagy is related to the ILK/PI3K signaling pathway (Zou et al., 2021). Wan et al. found that under the stimulation of CTS *in vitro*, the Hippo-YAP pathway perceives external stimuli and activates autophagy in human PDLCs (Wan et al., 2023). FOXO3, one of the FOXO family, is a transcription factor mediating the transcriptional activity of autophagy-related genes, including ATG7, LC3, and Bnip3. FOXO3 is sufficient and necessary to initiate autophagy. Activated AKT phosphorylates FOXO, blocking its nuclear translocation (Mammucari et al., 2007; Cao et al., 2013; Yu et al., 2019). Huang et al. found that the expression of LC3 and lncRNA FER1L4 is upregulated in compressed PDL of the mouse OTM model. Under orthodontic compressive force, upregulated FER1L4 inhibits the activation of the AKT/FOXO3 pathway, thereby enhancing the autophagic flux of human PDLSCs (Huang et al., 2021). However, the AKT activation plays different roles in mechanical force-mediated autophagy in osteocytes. Mechanical compressive force reduces the expression of ROCK and the phosphorylation of AKT in a time-dependent manner. The expression of TFE3 is increased, and more TFE3 is accumulated in the nuclei in MLO-Y4 cells subjected to compressive force. Further, treatment with ROCK inhibitor before compression suppresses the phosphorylation of AKT and increases the expression level of ATG7 and LC3-II (Li et al., 2020). These findings indicate that mechanical forces differentially regulate AKT activation in PDLCs and osteoblasts, both of which ultimately upregulate autophagy. Besides, mechanical tension suppresses AMPK protein expression as well as phosphorylation time-dependently, while inhibiting AMPK before applying tension in MLO-Y4 cells increases ATG7 and LC3-II expression levels, which indicates that mechanical tension triggers bone cell autophagy via AMPK signaling (Xu et al., 2021).

#### 4.2.2 Inflammatory factors

Orthodontic forces cause local hypoxia and fluid flow in PDL, thus initiating aseptic inflammatory cascades (Li et al., 2018). Some studies have reported that inflammation can affect OTM-induced autophagy. Blawat et al. found that autophagy of PDLFs is decreased with low concentration of IL-1 for 4h but increased with high concentration for 4h or under all assessed inflammatory conditions for 16 and 24h. Besides, the cell death rate is not affected by inflammatory stimulation for 4h but is increased for 16h. After 24 h, the low and high concentrations of IL-1β raise cell death numbers. These findings imply that the cell death and autophagy of PDLFs in response to inflammatory stimulation are dose and time-dependent (Blawat et al., 2020).

In summary, as an extracellular stimulus, mechanical force induces autophagy in PDLCs and osteocytes during OTM. Since PDLCs and osteocytes are important mechanical sensors of OTM, orthodontic force-induced autophagy may have a significant impact on tooth movement.

## 5 Autophagy regulate OTM

Some studies have confirmed that inhibiting or activating autophagy can lead to structural and cellular changes during OTM. Autophagy inhibitor 3-MA decreases the release of ATP and increases the cell death ratio in osteocytes (Zhang et al., 2018). Besides, 3-MA results in increased tooth movement distances, disordered arrangement of PDL, declining bone density, and reduced inflammatory cells in PDL tissues, but the opposite trend is observed in the treatment with autophagy activator rapamycin (Chen et al., 2019; Chen and Hua, 2021).

**Figure 3:**
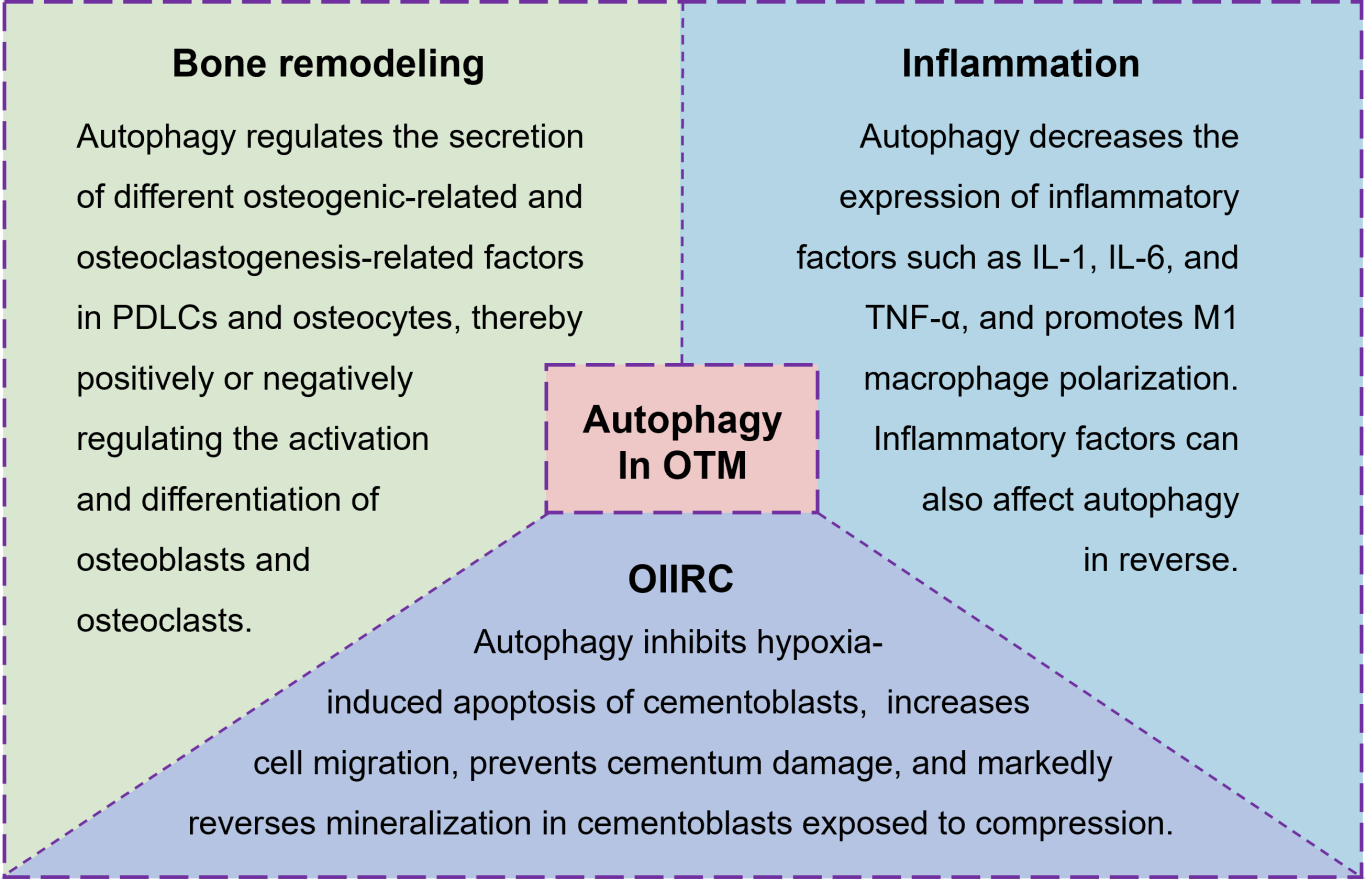
The role of autophagy in bone remodeling, inflammation, and complications during OTM.

Bone remodeling, including formation and absorption, is the foundation of OTM. These remodeling events cause inflammatory reactions in the periodontium, ultimately affecting the efficiency of OTM. Orthodontically induced inflammatory root resorption (OIIRR) is one of the inevitable complications of OTM (Yamaguchi and Fukasawa, 2021). We summarized the role of autophagy in bone remodeling, inflammation, and complications during OTM to evaluate its potential biological value in OTM (**Figure 3**).

### 5.1 Autophagy and bone remodeling

Osteoblasts and osteoclasts are the direct regulators of bone remodeling during OTM. It was found that autophagy is correlated with the activation and differentiation of osteoblasts and osteoclasts (Xu et al., 2020). Rapamycin inhibits the expression of osteoclast markers in PDL tissues and interferes with osteoclast recruitment in the mouse OTM model (Chen et al., 2019; Li et al., 2021a). Responding to the force applied, PDLCs, osteocytes, OBs, and OCs constitute an intricate network to modulate osteogenesis and osteoclastogenesis, which are mediated by the secreting proteins including various cytokines via autocrine or paracrine (Li et al., 2021b). Since autophagy is involved in the process of protein secretion (Kraya et al., 2015; Wang et al., 2019), some cell experiments *in vitro* have preliminarily confirmed the role of autophagy in the activation and differentiation of osteoblasts and osteoclasts, and revealed that some secreted proteins are involved in these networks.

#### 5.1.1 PDLCs-osteoblasts/osteoclasts

The expression of osteogenic-related factors is increased in human PDLSCs under orthodontic tension force loading. And significant differences are observed between the cells without tension force application and those with application at 1 h, 6 h, 12 h, and 24 h. Next, the expression of osteogenic-related factors (ALP, RUNX2, and OPN) is decreased after ATG7 knockdown or 3-MA treatment to inhibit autophagy in human PDLSCs, while rapamycin increases the expression of these osteogenic-related factors (Zheng et al., 2022). Similar results were reported by Shao et al. in human PDLSCs under CTS. The expression of osteogenic markers is upregulated after treatment with rapamycin, while downregulated after treatment with chloroquine (the autophagy inhibitor) (Shao et al., 2024). These results indicate that autophagy regulates the osteogenic differentiation of human PDLSCs induced by orthodontic tension.

RANKL is the essential cytokine required for osteoclastogenesis (Ono et al., 2020; Yasuda, 2021). 3-MA dramatically upregulates and rapamycin significantly downregulates the RANKL/OPG ratio in supernatants obtained from cultured PDLCs under compressive force for 6 hours, indicating that compressive force-induced autophagy might negatively regulate osteoclastogenesis by regulating the balance between RANKL and OPG expression via autocrine and paracrine mechanisms (Chen et al., 2019).

#### 5.1.2 Osteocytes-osteoblasts/osteoclasts

TRAP staining showed that more TRAP-positive osteoclasts accumulate on the alveolar bone surface of the distobuccal root of the maxillary first molar on the compression side of the mouse OTM model. IF staining revealed that increased LC3B-positive osteocytes are close to these osteoclasts (Li et al., 2020). Besides, ALP staining showed that the osteoblasts are increased on the alveolar bone surface of the distobuccal root of the maxillary first molar on the tension side, and IF showed a high number of LC3B-positive osteocytes near these osteoblasts (Xu et al., 2021). These findings suggest that the differentiation of osteoclasts and osteoblasts may be related to osteocytes.

Further *in vitro* cell experiments confirmed this hypothesis. The conditioned media from MLO-Y4 cells subjected to tension significantly enhance early osteogenesis in the pre-osteoblast cell line MC3T3-E1, and the effect is further enhanced when MLO-Y4 cells are treated with rapamycin to activate autophagy before the application of tension (Xu et al., 2021). OCN is well-known as a good marker for bone formation (Moriishi et al., 2020). Knockdown of ATG7 to inhibit autophagy suppresses ATP levels, OCN expression, and cell viability in osteocytes (Gao et al., 2023). FGF23 is a key regulator of bone and mineral metabolism and is secreted by osteoblasts and osteocytes (Ho and Bergwitz, 2021; Simic and Babitt, 2021). Tensile stress increases the expression and secretion of FGF23, which is further enhanced by rapamycin (Xu et al., 2021). Gao et al. also demonstrated similar results. CCF enhances FGF23 production and downregulates the expression of osteoclastogenesis-related factors (SOST, M-CSF, and RANKL). Meanwhile, the RANKL/OPG ratio is decreased significantly. The effect of CCF loading on the production of these cytokines is inhibited significantly in the presence of siATG7 to block autophagy (Gao et al., 2023). Further, the MC3T3-E1 cells and the osteoclast precursor cell line RAW 264.7 are cultured in conditioned medium collected from MLO-Y4 cells subjected to CCF. The promotion of osteogenic differentiation in MC3T3-E1 cells and inhibition of osteoclastogenesis and function in RAW 264.7 cells are significantly attenuated when osteocyte autophagy is inhibited by siATG7 (Gao et al., 2023). These studies suggest that mechanical loading-induced autophagy may regulate osteogenic differentiation and osteoclastogenesis by affecting the secretory ability of osteocytes.

However, Li et al. found that RAW264.7 cells cultured with conditioned medium from compressed MLO-Y4 treated with autophagy activator rapamycin exhibit large osteoclasts, and osteocytes could promote osteoclastogenesis via autophagy-mediated RANKL secretion under mechanical compressive force (Li et al., 2020). This discrepancy in different studies may be due to the secretion of different osteogenic and osteoclast-related factors regulated by mechanical force-induced autophagy.

Taken together, mechanical force-induced autophagy regulates the secretion of different osteogenic-related and osteoclastogenesis-related factors in PDLCs and osteocytes, thereby positively or negatively regulating the activation and differentiation of osteoblasts and osteoclasts. Given the complex roles of osteogenesis and osteoclastogenesis in bone formation, interfering with autophagy to affect bone formation may be a way to control OTM, and the mechanisms involved need to be clarified to achieve precise regulation.

### 5.2 Autophagy and inflammation

OTM leads to aseptic inflammation involving the release of inflammatory cytokines. The intensification of orthodontic force-induced inflammation can lead to aggravated periodontal destruction, ultimately affecting the effectiveness of OTM (Andrade et al., 2007; Maeda et al., 2007; Koyama et al., 2008). Extensive research has not yet fully elucidated how inflammation is regulated during OTM. The cytoplasmic clean-up function of autophagy is anti-inflammatory by default in any cell type capable of activating a cell-autonomous inflammatory response (Deretic, 2021). It has been reported that autophagy regulates pathogen invasion, the immune response, inflammation, and alveolar bone homeostasis of periodontal disease (Yang et al., 2021a). In addition to inflammation inducing autophagy, autophagy can also regulate inflammation of OTM.

In the tooth-movement model established in mice, animals were injected with 3-MA or rapamycin. 3-MA significantly increases the mRNA expression of inflammatory factors interleukin (IL)-1 and IL-6, as well as the protein expression of IL-6, and rapamycin decreases the mRNA expression of IL-1, IL-6, and TNF-α, compared with the OTM group (Chen and Hua, 2021). Besides, in human PDLFs under mechanical forces, the gene and protein expression of IL-6 is upregulated. The treatment with 3-MA to inhibit autophagy further enhances the effects of mechanical stimulation on IL-6 expression (Mayr et al., 2021).

In addition to inflammatory factors, autophagy also affects immune cells during OTM. Macrophages induce an inflammatory response by phagocytosing pathogenic microorganisms and releasing inflammatory cytokines. Besides, macrophages are precursors of osteoclasts and participate in bone remodeling. Thus, macrophages are regarded as one of the vital immune cells during the orthodontic process (Gordon, 2007; Feng and Teitelbaum, 2013; Nakatsu et al., 2014; Horwood, 2016). Autophagy activation is found in macrophages and osteoclasts in peri-dental tissues after orthodontic loading (Jacox et al., 2022). Mechanical forces modulate M1 macrophage polarization, which has been shown to be crucial in the bone remodeling and root resorption process during OTM (He et al., 2015b; He et al., 2015a). The number of M1 macrophages on the compression side of the PDL is decreased significantly after 3-MA injection into the rat OTM model. In human primary PDLSCs cultured *in vitro*, force-stimulated autophagy induces M1 macrophage polarization, which is related to the inhibition of AKT in macrophages (Jiang et al., 2021). However, Han et al. found that the application of force reduces autophagy in THP-1-derived macrophages as well as in the mouse OTM model. NLRP3 (the NOD-like receptor family pyrin domain-containing 3, an important sensor in the innate immune system forming the inflammasome) knockout significantly increases force-reduced autophagy in macrophages. The lack of NLRP3 inflammasome activation, which inhibits osteoclastogenesis during OTM, can be partially compensated for by autophagy inhibitors (Han et al., 2022). The discrepancies observed in the regulation of autophagy in macrophages may be attributed to the different mechanisms activated by mechanical forces. The role and regulatory mechanism of autophagy in immune cells during OTM still need to be carefully considered in future research.

In summary, there is an interaction between inflammation and autophagy during OTM. Force-induced autophagy inhibits the expression of inflammatory factors and promotes M1 macrophage polarization during OTM, and inflammatory factors can also affect autophagy in reverse. Since the relationship between inflammation and autophagy is of great significance for OTM, targeting autophagy may provide precise therapeutic targets for modulation of the inflammatory microenvironment in future orthodontic clinical treatment, so as to achieve more efficient and healthier tooth movement.

### 5.3 Autophagy and the complications of OTM

During OTM, local over-compression of the PDL may induce a hyalinization of the cementum, resulting in simultaneous cementum resorption in line with the removal of hyalinized tissue. This pathological process that causes substance loss from mineralized cementum and dentine is termed OIIRR (Yong et al., 2022). OIIRR, especially external root resorption, is a complication of orthodontic treatment, which can damage the dental bone and affect the long-term stability of teeth. Cementum regeneration is beneficial in the repair of root resorption (Zeichner-David, 2006; Oka et al., 2007; Weltman et al., 2010; Talic, 2011; Yang et al., 2015; Foster, 2017). Therefore, exploring the mechanism of cementum regeneration is of potentially great clinical significance for understanding, preventing, and treating OIIRC.

Cementoblasts are cells lining the tooth root surface, and play a critical role in the formation of the cementum matrix and tooth root repair (He et al., 2023). The decrease in periodontal oxygen partial pressure under orthodontic forces facilitates cementoblast apoptosis, which may be the mechanism of OIIRR (Korb et al., 2016; Matsuzawa et al., 2017). Xu et al. found that L-arginine enhances autophagy by upregulating the expression of SIRT1, thereby inhibiting hypoxia-induced apoptosis of cementoblasts. Next, it was confirmed in the rat OTM model that L-arginine reduces root resorption through SRIT1-mediated autophagy *in vivo*. L-arginine and SIRT1-mediated autophagy play essential roles in regulating root resorption, providing a potential new preventive and therapeutic strategy for root resorption (Xu et al., 2022).

In addition, the ability of cementoblasts to migrate to the site of resorption lacunae is the foundation for adhesion, proliferation, and differentiation to repair root resorption during OTM (Feller et al., 2016). In the murine cementoblast cell line, OCCM-30, autophagic flux and cell migration are reduced after compressive force loading, and cell migration is decreased with chloroquine treatment, while it is increased with rapamycin treatment. Besides, the expression of MMPs is downregulated under compressive force and is reversed after autophagy activation. These findings suggest that autophagy is inhibited and mediates the migration of cementoblasts under mechanical compression, and may be related to MMPs (Yang et al., 2021b).

The mineralization capacity of cementoblasts is also crucial for alleviating external apical root resorption during OTM (Iglesias-Linares and Hartsfield, 2017). Using the mouse root resorption model, the decreased expression of OCN, the mineralization-related protein, and LC3, the autophagy marker, is observed in cementoblasts on the compressed side *in vivo*. *In vitro*, cementoblasts subjected to compression loading also exhibit decreased mineralization capacity and inhibited autophagic flux. Treatment with rapamycin to activate autophagy markedly reverses mineralization in cementoblasts exposed to compression, and prevents cementum damage in the mouse root resorption model (Yang et al., 2023b).

Taken together, these studies elucidate the significant role of autophagy in regulating the function of cementoblasts. Thus, regulating autophagy in cementoblasts may be key to repairing root resorption during OTM. However, the underlying molecular mechanism remains unclear. Yang et al. found that autophagy restores cementoblast mineralization under compressive force through the Postn/β-catenin signaling axis (Yang et al., 2023b). Liu et al. observed that lncRNA-p21 is upregulated and modulates mineralization through autophagy in response to compressive force (Liu et al., 2022).

## 6 Conclusions and future perspectives

Orthodontic treatment requires a long duration of time and poses high risks of caries, external root resorption, and decreased patient compliance (Long et al., 2013). Thus, exploring the molecular mechanisms of OTM will help to identify potential targets to accelerate OTM and reduce possible side effects.

In this review, we summarize an update on the functions and mechanisms of autophagy in OTM. Using the mouse/rat OTM model *in vivo* and cells subjected to orthodontic forces *in vitro*, researchers have confirmed that mechanical forces induce autophagy in PDL and alveolar bone, especially in PDLCs and osteocytes, and multiple signaling pathways and inflammatory molecules are involved in this process. Then, we analyzed the multifaceted roles of autophagy in bone remodeling, inflammation, and complications during OTM to evaluate its potential biological value in OTM.

In view of the importance of autophagy in OTM, targeted activation of autophagy may shorten the duration of orthodontic treatment and reduce possible side effects. In animal OTM models, the treatment of the traditional autophagy inhibitor, 3-MA, increases tooth movement distances, disordered arrangement of PDL, and bone density, promotes inflammatory factors and inflammatory cells in PDL tissues, and accelerates OTM. However, treatment with rapamycin to activate autophagy results in the opposite trend during OTM and prevents cementum damage in the mouse root resorption model. Besides, other drugs such as Apocynin (Li et al., 2023), Sanhuang decoction (Yang et al., 2023a), and Strontium ranelate (Wu et al., 2023) have also been observed to affect autophagy in OTM.

Despite the widespread research on the role of autophagy in OTM, the exact mechanisms are not entirely investigated. Existing studies have preliminarily revealed that various signaling pathways as well as molecules are involved in these processes, and the autocrine and paracrine mechanisms of autophagy seem to influence the secretion of osteogenic-related and osteoclastogenesis-related factors. However, almost all of these experiments were conducted *in vitro* on cells. In the future, more *in vivo* experiments are needed in animal OTM models to reveal and validate the specific mechanisms of autophagy in OTM, thereby increasing the possibility of targeted autophagy in clinical orthodontic treatment.
